# Data of whole genome sequencing of five garden asparagus (*Asparagus officinalis*) individuals with the MinION nanopore sequencer

**DOI:** 10.1016/j.dib.2019.104838

**Published:** 2019-11-19

**Authors:** Daisuke Tsugama, Kaien Fujino

**Affiliations:** a Asian Natural Environmental Science Center, The University of Tokyo, Japan; b Research Faculty of Agriculture, Hokkaido University, Japan

**Keywords:** *Asparagus officinalis*, Gene cloning, Nanopore sequencing, Whole genome sequencing

## Abstract

Garden asparagus (*Asparagus officinalis*) is a perennial, dioecious crop. Genomic DNA samples were prepared from five *A. officinalis* individuals that differ in sex and phenotypes, and sequenced with the MinION nanopore sequencer. The obtained data were 1.5–5 Gb/sample, and the average read length was larger than 1.4 kb for all the samples. The resulting reads were mapped to the existing *A. officinalis* genome sequence. The existing *A. officinalis* transcript sequences were mapped to the MinION-derived reads. On the basis of these mapping results, flanking sequences of five partial gene fragments that previously had not been mapped to any region of the existing genome were determined by genomic PCR followed by Sanger sequencing. These sequences enabled to estimate the genomic positions of those five partial gene fragments. The MinION-derived data and the flanking sequences of the five gene fragments were deposited in the NCBI (National Center for Biotechnology Information) SRA (Sequence Read Archive) database and the NCBI Nucleotide database, respectively.

**Table:** Specifications Table

Subject	Plant Science
Specific subject area	Genomics of garden asparagus (*Asparagus officinalis*)
Type of data	Tables
How data were acquired	Whole genomes of *Asparagus officinalis* cultivars were sequenced with the MinION nanopore sequencer (type R9.4.1, Oxford Nanopore Technologies Ltd., UK (ONT)). Some of the *A. officinalis* gene fragments that had not been mapped to any region of the existing *A. officinalis* genome sequence were cloned by genomic PCR and confirmed by Sanger sequencing.
Data format	Raw Analyzed
Parameters for data collection	Female and male individuals of two *A. officinalis* cultivars, ‘Gold Schatz’ and ‘New Jersey 264’ (NJ264), and a supermale individual of ‘Mary Washington 500W’ were used for this study. They have been maintained in an open field in Hokkaido University for 10 years or longer. Genomic DNA for the sequencing was prepared from floral buds of those plants.
Description of data collection	The genomic DNA was sheared, end-repaired, and used for library construction. The MinION sequencing was run with the MinKNOW software (ONT). The resulting FAST5 files were converted to FASTQ files with the Albacore basecaller (version 2.1.3, ONT). The resulting read sequences were compared with the existing *A. officinalis* transcript sequences and the supermale genome sequence [[1], [2], [3], [4]] with the MegaBLAST aligner [5]. On the basis of this result, flanking sequences of five gene fragments whose genomic positions were unknown were determined by genomic PCR followed by Sanger sequencing.
Data source location	Hokkaido University Sapporo-shi, Hokkaido, Japan North latitude 43°07′ and east longitude 141°34′
Data accessibility	Repository name: NCBI (National Center for Biotechnology Information) SRA (Sequence Read Archive) and Nucleotide databases Data identification number: SRR9643835-SRR9643839 (SRA); MN311180-MN311184 (Nucleotide) Direct URL to data: https://www.ncbi.nlm.nih.gov/sra (SRA) https://www.ncbi.nlm.nih.gov/nuccore (Nucleotide)

**Table:** 

**Value of the Data** • These data add to the existing data of the Asparagus officinalis (garden asparagus) genome sequence, and facilitate cloning of genes and developing DNA markers of various *A. officinalis* cultivars • *A. officinalis* researchers and breeders will benefit from these data • These data can be used to modify incomplete parts of the existing *A. officinalis* genome data

## 1 Data

Samples and data regarding the MinION sequencing are summarized in Table 1. Raw data obtained from the MinION sequencing were deposited as FASTQ format in the NCBI SRA database (accession numbers: SRR9643835-SRR9643839, Table 1 “Data accessibility”). In a previous study, ∼2% of the *A. officinalis* gene fragments, which had been obtained from *de novo* assembly of RNA sequencing-derived reads [1,2], were not mapped to the existing *A. officinalis* genome sequence [4]. With the help of the MinION-derived data, flanking sequences of five of such gene fragments were cloned, and their genomic positions were estimated (Table 2). These sequences were deposited in the NCBI Nucleotide database (accession numbers: MN311180-MN311184, Table 1 “Data accessibility”).

**Table 1:** Summary of the MinION sequencing.

Cultivar, sex	Notes	NCBI BioSample ID^a^	NCBI SRA ID^a^	The number of reads	The number of bases
New Jersey 264, female	Cladodes have anthocyanins	SAMN12214960	SRR9643836	1363568	2032856420
New Jersey 264, male	Cladodes have anthocyanins	SAMN12214961	SRR9643835	1736516	3190967035
Gold Schatz, female	Cladodes have no anthocyanins; homeotic mutant [6]	SAMN12214958	SRR9643838	1411539	2464957402
Gold Schatz, male	Cladodes have no anthocyanins	SAMN12214959	SRR9643837	2296838	5536952756
Mary Washington 500W, supermale	Cladodes have anthocyanins	SAMN12214962	SRR9643839	669742	1525107676

a These are components of the NCBI BioProject PRJNA552649.

**Table 2:** Genes whose positions in the genome were estimated with the help of the MinION data.

Gene name^a^	NCBI Nucleotide ID	Corresponding position in the genome^b^	Closest homolog in Arabidopsis
Aspof_comp39872 _c1_seq1	MN311180	Chromosome 2, 3191600-3194220	AT2G39350.1 (ABC-2 type transporter family protein)
Aspof_comp56646 _c4_seq1	MN311181	Chromosome 1, 132224408-132225250	AT4G24660.2 (Homeobox protein 22)
Aspof_comp57098 _c0_seq1	MN311182	Chromosome 9, 62811727-62814775	AT3G48800.1 (Sterile alpha motif (SAM) domain-containing protein)
Aspof_comp57295 _c1_seq1	MN311183	Chromosome 5, 14627566-14628935	AT3G51550.1 (Malectin/receptor-like protein kinase family protein)
Aspof_comp59943 _c2_seq1	MN311184	Chromosome 3, 973061-973883	AT5G15210.1 (Homeobox protein 30)

a These are names of the transcripts (i.e., contigs) generated by *de novo* assembly of RNA sequencing-derived reads [1,2].

b The positions correspond to the regions with sequential undetermined (‘N’) bases in the existing *A. officinalis* genome sequence.

## 2 Experimental design, materials, and methods

*A. officinalis* plants including five individuals used in this study have been maintained for 10 years or longer in an open field in Hokkaido University. Genomic DNA was prepared from their floral buds with the DNeasy Plant Mini kit (Qiagen, Germany). DNA shearing, end-repair, dA-tailing and adapter ligation for library construction for the MinION sequencing as well as library loading into the MinION flow cell (R9.4.1, ONT) were performed with Ligation Sequencing Kit 1D (ONT), g-TUBE (Covaris, USA), NEBNext Ultra II End Repair/dA-Tailing Module (New England Biolabs, USA (NEB)), Blunt/TA Ligase Master Mix (NEB) and Library Loading Bead Kit (ONT), according to ONT's instructions for 1D Lambda Control Experiment. The sequencing run was performed with the MinKNOW software with the live basecalling option disabled. For each library (i.e., sample), a new flow cell was used, and the run time was 48 hours. The resulting FAST5 files in the “pass” folders, which correspond to sequences with high quality scores, were converted to FASTQ files with the Albacore basecaller (version 2.1.3, ONT). The resulting FASTQ files were concatenated according to samples, and deposited in the NCBI SRA (Sequence Read Archive) database (accession numbers are presented in Table 1).

The reads derived from the MinION sequencing were mapped to the existing *A. officinalis* genome sequence, which derived from a supermale individual (NCBI RefSeq accession: GCF_001876935.1) [3], with the MegaBLAST aligner in the BLAST + suite [5] with default parameters. The putative *A. officinalis* transcript sequences that were derived from RNA sequencing [1,2] and that were not mapped to the above-mentioned genome sequence [4] (‘orphan genes’) were mapped to the MinION-derived reads with MegaBLAST with default parameters. Flanking sequences and genomic positions of five randomly chosen orphan genes were estimated with those MegaBLAST results. These orphan genes and their flanking sequences were amplified by PCR using the male NJ264 plant-derived genomic DNA as the template and the primer pairs listed in Table 3. Sequences of the resulting PCR products were determined by Sanger sequencing [7], and deposited in the NCBI Nucleotide database (accession numbers are presented in Table 2).

**Table 3:** Primers used to clone the genomic regions of *A. officinalis* gene fragments that had not been mapped to the existing *A. officinalis* genome sequence.

Target gene	Primer sequence (5' > 3′)	Annealing site^a^
Aspof_comp39872_c1_seq1	TCCCTCCAATTCACTCACCATTTGAACATC	Chromosome 2, 3191383-3191412
Aspof_comp39872_c1_seq1	TTAGATTTAGATTGCATCATAACCACCTAC	Chromosome 2, 3194827-3194798
Aspof_comp56646_c4_seq1	AGTGAGAACAAGTAGAGCAAACTGAGGCAG	Chromosome 1, 132224305-132224334
Aspof_comp56646_c4_seq1	ACTGCATGCACATACATAGATGCAGTAGAG	Chromosome 1, 132225392-132225363
Aspof_comp57098_c0_seq1	GAGATGACTTTGAGTTGCTACTTCGACATC	Chromosome 9, 62810323-62810352
Aspof_comp57098_c0_seq1	TTTGGAGGTCAAGTACGACTTCTAAAAGCC	Chromosome 9, 62815221-62815192
Aspof_comp57295_c1_seq1	TCTCGGCTGTCTCGACGAACTTCTTGAAGC	Chromosome 5, 14626818-14626847
Aspof_comp57295_c1_seq1	AGAAACATGAGATCTTACATGGGACATGTG	Chromosome 5, 14629427-14629397
Aspof_comp59943_c2_seq1	CACATACAACTTCAGTTTGAAGCCAAGATC	Chromosome 3, 972084-972113
Aspof_comp59943_c2_seq1	CTGAGACAGTTACAACCGATTATCAGGATG	Chromosome 3, 974605-974576

a If X > Y in the pattern “X–Y” (i.e., in the second, fourth, sixth, eighth and tenth rows), corresponding primers anneal in the reverse orientation.
